# Hair Cortisol and Perceived Stress—Predictors for the Onset of Tics? A European Longitudinal Study on High-Risk Children

**DOI:** 10.3390/biomedicines11061561

**Published:** 2023-05-27

**Authors:** Josefine Rothe, Judith Buse, Anne Uhlmann, Benjamin Bodmer, Clemens Kirschbaum, Pieter J. Hoekstra, Andrea Dietrich, Veit Roessner

**Affiliations:** 1 Department of Child and Adolescent Psychiatry and Psychotherapy, Technische Universität Dresden, 01307 Dresden, Germany; 2 Department of Psychology, Institute of Biopsychology, Technische Universität Dresden, 01307 Dresden, Germany; 3 Department of Child and Adolescent Psychiatry, University of Groningen, University Medical Center Groningen, 9713 GZ Groningen, The Netherlands; 4 Accare Child Study Center, 9723 HE Gronigen, The Netherlands

**Keywords:** tic disorder, physiological stress marker, hair cortisol, perceived stress, onset of tics

## Abstract

Some retrospective studies suggest that psychosocial stressors trigger the onset of tics. This study examined prospective hypothalamic–pituitary–adrenal (HPA) axis activity and perceived stress prior to tic onset. In the present study, 259 children at high risk for developing tics were assessed for hair cortisol concentration (HCC) and parent-on-child-reported perceived stress four-monthly over a three-year period. We used (i) generalised additive modelling (GAM) to investigate the time effects on HCC (hair samples *n* = 765) and perceived stress (questionnaires *n* = 1019) prior to tic onset and (ii) binary logistic regression to predict tic onset in a smaller subsample with at least three consecutive assessments (six to nine months before, two to five months before, and at tic onset). GAM results indicated a non-linear increasing course of HCC in children who developed tics, and a steady HCC course in those without tics, as well as a linear-increasing course of perceived stress in both groups. Logistic regression showed that with a higher HCC in hair samples collected in a range of two to five months before tic onset (which refers to cortisol exposure in a range of four to eight months), the relative likelihood of tic onset rose. Our study suggests increased stress prior to tic onset, as evidenced by higher HCC several months before tic onset.

## 1. Introduction

Sudden, recurrent, rapid, nonrhythmic movements such as eye blinks or vocalisations are common phenomena in childhood, observed in up to one-fifth of children in the general population [1,2,3]. Chronic tic disorders (CTDs), characterised by motor and/or vocal tics lasting at least 1 year affect approximately 1–3% of children worldwide [4,5]. While we previously identified certain mental and behavioural problems (such as conduct problems, compulsions, or emotional problems) as precursors of tic onset [6], early-life psychosocial stress is an environmental risk factor suspected to trigger the onset of tics. However, to date, studies examining psychosocial stress (i.e., the exposure to adverse life events or the subjective appraisal of situations as being overwhelming or very demanding) prior to the onset of tics are limited to retrospective assessments of stressful life events [7,8,9,10], while prospective studies including physiological markers of psychosocial stress (such as cortisol) and perceived stress are lacking. 

Individuals experience psychosocial stress when they perceive an imbalance between demands and resources [11]. Psychosocial stress manifests physiologically as an activation of the hypothalamic–pituitary–adrenal (HPA) axis, a multi-step biochemical pathway that in turn regulates the release of cortisol, the main stress hormone. Studies on other psychiatric disorders (e.g., postpartum depression and posttraumatic stress disorder) revealed cumulative cortisol levels (measured by hair cortisol concentration, HCC, referring to the stress level over the last two to three months) as a predictor of symptom onset [12,13,14]. Furthermore, studies indicated that the relationship between symptom severity and hair cortisol is non-linear [15,16], which may also apply to the onset of symptoms.

So far, evidence about the relationship between tics and cortisol levels is limited, and often, the study design did not match the complex interplay of many associated factors. For example, there is evidence that children and adults with CTDs show a stronger activation of the HPA axis (i.e., higher cortisol levels) compared to healthy controls when exposed to acute psychosocial stressors [17,18,19]. Importantly, the mentioned studies used short-term assessments of cortisol level changes that occur over the course of minutes to hours and are therefore not suitable to evaluate the activation of the HPA axis over a longer period (months). To date, there has only been one study in children and adolescents with CTDs examining HCC as a retrospective longer-term assessment of HPA axis activity [20]. In this study by our group, a weak-to-moderate relationship between tic severity and perceived stress was found, but no relationship between tic severity and HCC and no difference in HCC between affected and unaffected children. To date, there have been no prospective data on HCC and perceived stress as a possible precursor of tic onset, despite being of clinical interest, as future preventive efforts can be directed towards this.

Thus, the main aim of the present study was to examine whether HCC and perceived stress are precursors of tic onset. It is the first study ever assessing the effect of HCC and perceived stress on the onset of tics in a prospective manner. In the present study, over a three-year period, we measured HCC (a marker referring to the cumulative cortisol level indicating prolonged stress over the last two to three months) and parent-on-child-reported perceived stress (covering the previous month) every four months and at the time of tic onset in children at heightened risk of developing tics (siblings of children with a CTD, who themselves had no tics before or at study entry [21]). We compared the participating children who developed tics during the course of the study (Onset+) with those who did not (Onset−). First, we examined the non-linear time effects on levels of stress (HCC and perceived stress) and whether they were different between Onset+ and Onset− children (Onset±) using generalised additive modelling over a three-year course. Next, only children with at least three consecutive assessment points were investigated. That is, it was of interest whether levels of stress (HCC and perceived stress) within the three separate time periods (i.e., six to nine months before, two to five months before, and at tic onset) would predict the likelihood of tic onset. 

We expected a non-linear course of HCC and perceived stress before tic onset. We further hypothesised that HCC and perceived stress predict tic onset, with a higher HCC and perceived stress before tic onset. 

## 2. Materials and Methods

### 2.1. Study Design and Participants

Our data are part of a longitudinal European cohort, the European Multicentre Tics in Children Studies (EMTICS), designed to identify the genetic and environmental risk factors of CTDs [22]. The ONSET arm of EMTICS investigates the association between environmental and genetic factors and the onset of tics in first-degree relatives (siblings) of individuals with a CTD. This study arm comprises 259 children aged 3–10 years (at the time of baseline visit) who are siblings of an individual with a CTD, who themselves had no tics before or at study entry. A total of 61 children developed tics during the three-year course of the study (see [6]). The presence of comorbidities such as attention-deficit/hyperactivity disorder (ADHD), obsessive–compulsive disorder (OCD), and trichotillomania were assessed in a clinical interview according to DSM IV-TR criteria. Family demographic data were also recorded. Per protocol, OCD and trichotillomania were exclusion criteria in the ONSET cohort. Yet, some protocol breaches occurred, which resulted in 24 individuals with OCD and four individuals with trichotillomania still entering the ONSET arm. For the present HCC study, these children were excluded (consistent with our previous HCC study [20], and given the potential association between HCC and OCD), resulting in a sample of 231 (out of 259) children (*n* = 54 with and *n* = 177 without tic onset; this sample included 19 sibling pairs). However, at least three consecutive assessment points with both available HCC and perceived stress within designated time windows (see statistical analyses) were available for 72 children only (*n* = 13 with and *n* = 59 without tic onset; this sample included seven sibling pairs; note that there was only one child with tic onset and OCD not included in this study). Children and their families were recruited from 16 child and adolescent psychiatry, neurology, and paediatric neurology outpatient clinics across Europe with additional study advertisement through patient organisations and other health professionals (for more details, see [22]). 

The study was approved by the Institutional Review Board of each participating centre. Parents and their children provided written informed consent and assent as appropriate according to local ethical regulations. While the travel costs of participating families were reimbursed, no additional compensation was paid.

### 2.2. Study Procedure 

At the baseline visit, parents were explained all possible symptoms indicative of a possible onset of tics and were instructed to communicate any possible sign of tic onset to the study centre as soon as possible. The baseline visit was followed by planned four-monthly alternating telephone interviews (comprising a review of weekly diaries about possible symptom onset and clinical evaluations of possible tic onset) and site visits (comprising clinical evaluations and collection of hair). Thus, over the three-year study period, as per protocol, children were evaluated every two months, allowing for assessments two weeks earlier or later than according to the visit schedule. Nevertheless, there were also larger time gaps between the two assessments due to individual scheduling difficulties and missed visits. In addition to the scheduled assessments, parents were asked to get in touch with the clinical centre in case of a suspected onset of tics, which could lead to an expedited tic onset visit, thus shortening the planned inter-visit period.

The onset of tics was defined as the first occurrence of any sudden, rapid, recurrent, nonrhythmic, involuntary motor movement and/or vocalisation noticed on at least three consecutive or separate days within a period of three weeks. If a possible tic onset was identified through a telephone interview, a site visit followed within one week (or at the earliest opportunity) for a clinical evaluation to confirm or exclude the parent-observed tic onset and for an assessment of levels of stress (HCC and perceived stress) [see Figure S1 in the Supplementary Material for the study workflow].

### 2.3. Measures

Hair cortisol concentration (HCC): HCC was measured as a physiological marker of longer-term stress. It was extracted from the two to three most proximal centimetres of the gathered scalp hair strains, taken approximately two cm below the cranial bone. This indicates the cortisol exposure over the past two to three months, as the growth rate of hair is approximately one cm per month [23]. The analysis procedure followed a protocol described in detail by Dettenborn and colleagues [24] with two changes described previously by our group [20]. From siblings of individuals with CTDs, we collected *n* = 894 hair samples from the baseline until the tic onset visit or throughout participation (with no tic onset). Unwillingness to provide a hair sample or too short of hair were the most common reasons why hair samples were not collected. Due to insufficient weight (<4 mg) or insufficient length (<2 cm) for laboratory analyses, 120 hair samples were excluded. Another nine hair samples were excluded as they were HCC outliers (≥3 SD/28.8 pg/mg). This resulted in a total hair sample size of 765 (128 of them from Onset+ children). 

Perceived Stress Scale (PSS-10): As a measure of perceived stress, the parent-on-child version of the well-established 10-item self-report version of the Perceived Stress Scale [25] was used. A parent rated the degree to which life situations over the past month were experienced as stressful by the child on ten questions with a five-point response scale (range 0–40, higher scores indicated more perceived stress). The questions and scales of the parent-on-child version were the same as in the self-report version (e.g., “In the last month, how often has your child been upset because of something that happened unexpectedly?”). The 10-item self-report version was found to be reliable and valid (Cronbach’s alpha 0.74–0.91; criterion validity *r* = 0.70) [26]. The relation between the PSS-C and the PSS-P is strong (*r* = 0.51–0.59, [27]). We only used the parent-on-child version, since all children were under or at the age of ten, and child-report measures are available only from age 11 years. A total of 1019 PSS-10 questionnaires (190 of them from Onset+ children) were collected. 

Parental education level: For the parental education level, the highest completed level of education of the mother and/or the father was captured. If information was available from both parents (included as a parent: biological parent, partners of divorced parents, or adoptive parent), the higher educational level was used. If information was only available from one parent, their education level was used. It was rated as follows: 1 = under seven years of schooling, 2 = Junior High School/7th–9th grade, 3 = General Certificate of Secondary Education or high school diploma, 4 = A levels or two-year college degree, 5 = four-year college/university degree, and 6 = post-graduate/graduate/professional degree.

### 2.4. Statistical Analyses

Group differences in age, time (weeks) between visits, HCC, and PSS-10 were examined by independent *t*-tests. To identify differences in sex, the highest education of parents, and the presence of ADHD between the two study groups, we conducted chi-square tests. False discovery rate (FDR) correction [28] was applied for comparisons of HCC and PSS-10 to correct for multiple testing. Effect sizes were calculated using Phi and Cramer’s V for chi-square tests and Cohen’s d for independent *t*-tests [29]. 

We applied generalised additive models (GAM) for HCC and PSS data separately, which can be used to determine complex non-linear regression effects [30] by automatically determining the optimal combination of non-linear basis functions [31]. For the present data (*n* = 54 with and *n* = 177 without tic onset), a GAM offers a suitable method to examine the time course of HCC, despite the missing values (by setting each hair sample and PSS measure as a separate case), and is not limited to linear assumptions. To model the means of HCC and PSS-10 as (potentially) non-linear functions of time and test for a significant interaction effect between Onset± (group effect of Onset+ and Onset−) and time, i.e., if the patterns across time before tic onset varied depending on Onset±, we implemented GAMs for the two outcomes, HCC and PSS-10, with the following form:

g(outcome) = α + βX + f_1_(time, by = Onset±)

where α is the intercept, β is the vector of parameters associated with the set of explanatory variables X (Onset±, Sex, and Age), and f_1_ is a smooth function for time (for Onset+ children: weeks to Onset; for Onset− children: weeks to last visit) by Onset± (to test the interaction effect between Onset± and time). The smooth functions are associated with estimated degrees of freedom (EDF) indicating a linear (EDF = 1) or non-linear (EDF > 1) relationship with the related p-value indicating whether the shape and direction of the effect is certain.

In addition to the GAMs, binary logistic regression analyses were performed to investigate whether levels of stress (HCC and PSS-10 as the independent variables) in the months before and at tic onset had an effect on the likelihood to develop tics, with Onset± as the dependent variable. For this purpose, we only selected children with available HCC and PSS-10 on at least three consecutive study visits (*n* = 13 with and *n* = 59 without tic onset). As different time spans were covered by the HCC (the past two to three months) and PSS-10, separate analyses were run for HCC and PSS-10 (past month). For the Onset− children, it comprised the first three visits every four months, including the baseline visit. For the Onset+ children, it comprised the individual’s last three visits, including the tic onset visit. As the hair sample at tic onset indicates the cortisol exposure over the past two to three months, the previous hair sample for this analysis had to be collected at least two months before tic onset and at the most six months before (staying within the four-month assessment rhythm). Hence, the study visits in the Onset+ children refer to three time periods (T1: representing a time range of six to nine months before tic onset, T2: representing two to five months before onset, and T3: at tic onset). Binary logistic regression analyses were run for HCC and PSS-10, using the enter method with one block (includes the respective stress measures, HCC or PSS-10, at the three time points), and showing 95% Confidence Intervals. We a priori tested the independent variables (HCC and PSS-10 over the three time periods) to verify that the assumption of the linearity of the logit was fulfilled and that there was no multicollinearity. 

Due to the skewed distribution of HCC and PSS-10 data, we applied a Box–Cox transformation [32] with −0.50 (a reciprocal square root transformation) as the best-fitting lambda for HCC and 0.70 for PSS-10. Transformed HCC and PSS-10 values were used for independent *t*-tests and GAMs. The mgcv package [31] working in the RStudio 2022.07.2 (R 4.1.2) environment was used to conduct the GAMs, while SPSS 28 was used for all other analyses.

## 3. Results

### 3.1. Description and Characteristics of the Total Study Sample and Subsample Used for Analyses

Sample characteristics and the results of chi-square tests and *t*-tests are displayed in Table 1 regarding the children included in the present study and the smaller subsample used for the binary logistic regression analyses (for more details on sample selection, see Table S1 in the Supplementary Material). A total of 5 of the 177 Onset− children and 6 of the 54 Onset+ children reported psychotropic medication use at some point during study participation. 

We found no significant differences regarding parental education, ADHD, or age between Onset+ and Onset− children (see Table 1). A small effect was found for sex between the two study groups (Onset+ children were more likely to be male).

According to the study protocol, further evaluations stopped after tic onset, while Onset− children were evaluated for up to three years. Therefore, the duration of study participation (for Onset+ this equates to the time until onset) differs significantly between the groups (see Table 1). Since in the smaller subsample, the two included visits before tic onset could be within a time span of four months (T1: visit occurred six to nine months before onset; T2: visit occurred two to five months before onset), it was checked whether the time between the two measures differed between the groups. The mean number of weeks between the visits used for the analyses did not differ between the two groups (see Table 1). 

Table 2 shows both stress measures in the smaller subsample used for binary logistic regression. HCC at T2 was significantly higher in the Onset+ children compared to the Onset− children, while all other stress measures (HCC and PSS-10 at T1 and T3 and PSS-10 at T2) did not differ significantly between the groups.

Across the total study sample and all measurements, there was no significant correlation between HCC and PSS-10, *r* (726) = −0.02, *p* = 0.55.

### 3.2. Non-Linear Time Effects

**Figure 1:**
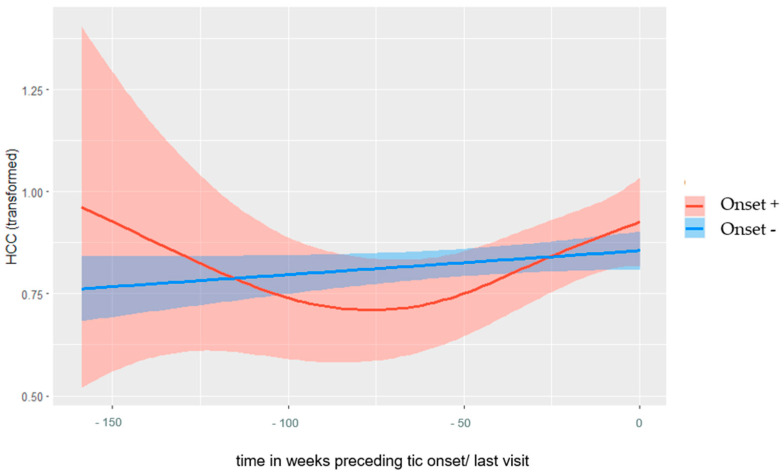
Estimated curves for HCC (of 128 Onset+ hair samples and 637 Onset− hair samples) during study participation in Onset+ and Onset− children, with shading of the 95%-level confidence region. Note. The Onset+ end point was at tic onset and the Onset− end point was the last visit.

As seen in Figure 1, Onset+ children showed a different pattern of HCC across time (time to Onset) than Onset− children (time to last visit). While Onset− children showed rather steady HCC values, Onset+ children showed lower levels one to two years before onset, followed by an increase above the level of Onset− children. However, for Onset+ children in the study, the confidence interval longer than two years before onset was very large (due to the low sample size of hair samples collected more than two years before tic onset). The GAM estimation indicated, through the EDF score of 2.25 for the smooth term of Onset+ specific time effects (weeks to onset/last visit), a non-linear course of HCC for Onset+ children. 

However, the smooth term did not reach the significance level of <0.05 (*p* = 0.07), meaning there was some complexity in the HCC-course of Onset+ children but also some uncertainty about the shape and direction of the effect. In contrast, the EDF score of 1 for the smooth term of Onset− specific time effects (time to last visit) indicated a linear course of HCC for Onset− children, but even this smooth term does not reach the significance level of <0.05 (*p* = 0.08). The difference between the estimated smooth terms did not reach a significance level of <0.05 (EDF = 1.00, *p* = 0.08), meaning that the difference between the HCC pattern in Onset+ and Onset− children did not reach significance (due to the large confidence interval). 

**Figure 2:**
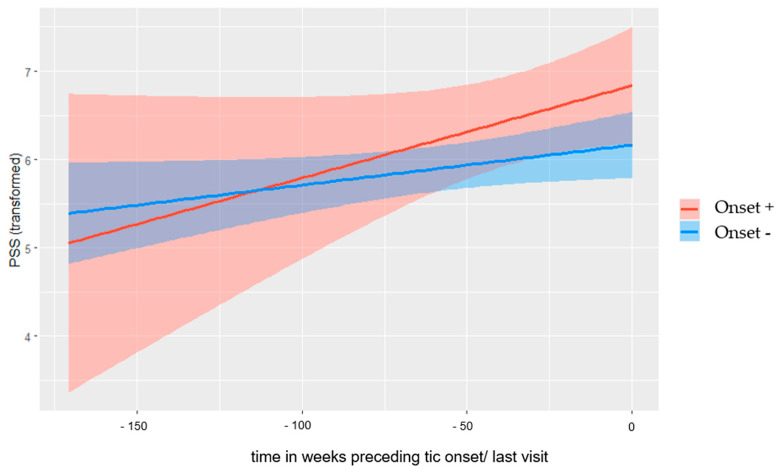
Estimated curves for PSS-10 (of 190 Onset+ questionnaires and 829 Onset− questionnaires) during study participation in Onset+ and Onset− participants with shading of the 95%-level confidence region.

Both groups showed a linear pattern of PSS-10, indicated by the EDF score of 1, for the smooth term of Onset± specific time effects (weeks to onset/last visit). As can be seen in Figure 2, higher perceived stress was implied in Onset+ children in the months before onset. Again, the confidence interval longer than two years before onset in Onset+ children was large and both smooth terms did not reach the significance level of <0.05 (Onset+ *p* = 0.09; Onset− *p* = 0.05). The difference between the estimated smooth terms was also not significant (EDF = 1.00, *p* = 0.06), meaning that the difference between the PSS-10 pattern in Onset+ and Onset− children did not reach significance (due to the large confidence interval). Furthermore, the linear term of age was significant (*p* = 0.04), indicating a linear relationship between age and PSS-10.

All coefficients of the generalised additive models can be seen in Table S2 in the Supplementary Material. Repeating the GAM for HCC and PSS-10 without sibling pairs (by either excluding the Onset- children in Onset+/Onset− pairs, or the younger and male children in Onset−/Onset− pairs) did not change the patterns of the smooth terms (see Table S3 in the Supplementary Material). An additional repetition of the analysis with a case-control matched sample (without sibling pairs) also did not change the patterns of the smooth terms (see Table S4 in the Supplementary Material).

### 3.3. Effects of Stress up to Twelve Months before the Onset of Tics on the Likelihood of Developing Tics

To examine whether HCC and PSS-10 levels before the onset of tics increased the likelihood of tic onset, we conducted binary logistic regression analyses with tic onset as the dependent variable and HCC (for HCC model) or PSS-10 (for PSS-10 model) at T1, T2, and T3 as independent variables.

The assumption of the linearity of the logit was given for all independent variables (log interaction term: HCC at T1 *p* = 0.36; HCC at T2 *p* = 0.80; HCC at T3 *p* = 0.23; PSS at T1 *p* = 0.71; PSS at T2 *p* = 0.36; and PSS at T3 *p* = 0.74) and there was no multicollinearity (variance inflation factors: HCC at T1 = 1.1; HCC at T2 = 1.14; HCC at T3 = 1.04; PSS at T1 = 2.5; PSS at T2 = 3.3; and PSS at T3 = 2.1). 

The overall binary logistic regression model for HCC was significant, χ^2^(3) = 10.13, *p* = 0.02. The model explained 21.5% (Nagelkerke R^2^, which is equivalent to an effect size of *f*^2^ = R^2^_NK_/(1 − R^2^_NK_) = 0.27 and thus a medium effect) of the variance in tic onset with an overall correct prediction rate of 84.7%. HCC at T2 was a significant predictor of tic onset at T3, Wald = 5.47, *p* = 0.02. With each 1 pg/mg higher HCC at T2, the relative likelihood of tic onset at T3 rose by 30.1% (CI 1.04–1.62). The other two predictors were not significant (HCC at T1: Wald = 0.22, *p* = 0.64; HCC at T3: Wald = 0.02, *p* = 0.88). For PSS-10, the binary logistic regression model was not significant, χ^2^(3) = 0.83, *p* = 0.84.

## 4. Discussion

This study is the first to prospectively examine the three-year course of hair cortisol and perceived stress and whether levels of stress (up to one year prior to onset) are predictors of the onset of tics in children with a heightened risk of developing tics (i.e., siblings of individuals with a CTD). The results of the generalised additive model (GAM) suggest a non-linear increasing pattern of HPA axis activity before tic onset, although there is some uncertainty about the shape and direction of the effect. A visual inspection of the model implies higher HCC levels in the five months before tic onset compared to Onset− children. In comparison, Onset− children showed a rather steady HPA axis activity. Notably, for hair samples collected within a range of two to five months before tic onset (which refers to the cortisol exposure in a range of four to eight months before tic onset), with each 1 pg/mg higher HCC, the relative likelihood of tic onset rose by 30%. Similarly, HCC levels in the Onset+ group were significantly higher two to five months before tic onset compared to the Onset− group. This is in line with the non-linear increasing pattern of HPA axis activity before tic onset identified by GAM. For perceived stress, the GAM results suggest a linear increasing pattern for both Onset+ and Onset− children, and a visual inspection of the model indicates higher perceived stress in Onset+ children in the five months before onset. However, for the three separate time periods of the binary logistic regression analyses (T1: six to nine months, T2: two to five months before, and T3: at onset), we did not find any effects of perceived stress on the likelihood of developing tics.

Given the results of the binary logistic regression analysis regarding HCC, it is rather surprising that no effect of perceived stress on the likelihood of developing tics was found. It was also unexpected that there was no association between HCC and perceived stress. In this context, however, it must be considered that results on the relationship between HCC and psychosocial stress in humans are also inconsistent (for a meta-analysis see [33]), which could relate to different types of measured psychosocial stress (e.g., daily/weekly hassles, adverse life events), different reporting perspectives (e.g., self, parent, and partner), and the time lag between physiological and psychosocial measures. For example, in a study of 37 adult couples, self-reported weekly hassles predicted HCC with a time lag of about four weeks. Meanwhile, the authors found no association between HCC and partners’ reports of weekly hassles (frustrating demand of everyday transactions) [34], as well as self and partner reports on the perceived stress scale or chronic stress scale, even with a time lag of about four weeks [35]. This underlines the complexity of the relationship between HCC and psychosocial stress—with HCC as a retrospective physiological marker of longer-term stress in terms of time and perspective.

Therefore, besides the small sample size for the binary logistic regression analyses with limited power for a general conclusion, we see two further limitations in our study design that might explain our findings of the medium effect of HCC and not of perceived stress on tic onset. First, different time spans were covered by HCC and the PSS-10. In the present study using four-monthly assessments, HCC represented the cortisol level covering the past two to three months prior to its assessment, whereas the PSS-10 reflected the perceived stress of the last month. Stressful episodes longer than one month but less than three months ago could thus have led to increased HCC but may not have been captured by the PSS-10. In other words, the PSS-10 may not have indicated the chronicity of stress needed to be related to tic onset. Ideally, both stress measurements (HCC and PSS-10) should have been carried out more frequently, but compliance would have probably decreased considerably if hair samples were taken even more frequently (e.g., every eight weeks). However, instead of carrying out the HCC measurements more frequently, the HCC could also be analysed in much smaller increments. For instance, the samples could be subdivided into 1 cm sections, each capturing ~1 month of cortisol. For future studies, it could, therefore, be helpful to combine cortisol measures of hair (subdivided into 1 cm sections), saliva, and sweat (measured by wearables) as physiological measures of stress and to capture perceived stress by a mobile application every four weeks. Second, children’s perceived stress was measured by parental ratings. Consequently, episodes perceived as stressful by the children may not have been identified if parents judged them as less severe or not stressful at all. For example, parents may judge a tic exacerbation of their CTD child as stressful for this child. However, a tic exacerbation could also be perceived as stressful for the unaffected sibling. Unfortunately, an insufficient number of child ratings was available for analysis. 

Even if speculative, the present results of higher HCC before tic onset could point to an overactivation of the HPA axis, perhaps as an inflammatory stress-related phenomenon, such as increased proinflammatory cytokine levels that enhance activation of the HPA axis (e.g., the overproduction of the corticotropin-releasing hormone, CRH), reflecting an enhanced response to stress [36]. However, caution is warranted for interpretation as further factors (e.g., emotional problems, compulsions, or oppositional symptoms [6]) could lead to changes in HPA axis measures before tic onset, rather than the association being mediated by (psychosocial) stress. However, there is growing evidence of aberrant proinflammatory cytokine levels in individuals with CTDs and indications that cytokine levels in patients with CTDs are related to the severity of tics [37,38,39]. Furthermore, a stronger HPA axis activation in terms of elevated CRH levels in the cerebrospinal fluid has been found in individuals with CTDs compared to healthy controls (+28%) and in those with OCD (+31%) [19]. Both animal and human studies (e.g., [40,41]) have shown that CRH (which increases as a response to stress) modulates dopamine release, and dopamine, for its part, affects motor circuits in the brain, which can potentially lead to hyperkinetic symptoms such as tics (e.g., [42]). Although dopamine is not the only neurotransmitter involved in the cortical–striatal–thalamic–cortical circuits, it is further involved in immune-mediated mechanisms [36,43,44]. A hyper-dopaminergic state has been proposed in CTDs to exert immunomodulatory effects, e.g., upregulated proinflammatory immune responses, hence, elevated physiological stress responses, and thereby enhancing tic symptoms [36,44]. 

The suggestion of overactivation of the HPA axis is consistent with growing evidence about multimodal hypersensitivity to external stimuli related to altered central sensory processing in individuals with CTDs [45]. For example, a large proportion of individuals with CTDs report heightened sensitivity to external stimuli, and while the detection threshold is unchanged compared to healthy controls [46], sensorimotor gating is deficient [47]. Similar processes may also be involved in relation to tic onset. 

Strikingly, in a previous study by our group, we found no relationship between tic severity and HCC at baseline in individuals with CTDs and also no difference in HCC between individuals with CTDs and unaffected siblings [20]. 

Additional limitations should be acknowledged. In the present study, siblings of children with CTDs who are at increased risk for developing tics were examined. Therefore, results from this study suggest that higher HCC several months before tic onset, at least in those with genetic risk factors, does play a role in the onset of tics, yet the results cannot be generalised to non-familial CTDs. While, in line with expectations, a group size of 61 out of 259 children with tic onset within the three-year study period was achieved, only 13 of them had conjoint valid hair and perceived stress samples at tic onset and at two previous visits before tic onset. Although generalised conclusions from a sample of 13 Onset+ children are limited, the patterns of HCC and PSS-10 identified by the GAMs in the larger sample point in the same direction as the results of the binary logistic regression in the small sample. Furthermore, the study design does not allow for causal interpretations, as increased stress levels may be related to increased psychopathological symptoms found prior to tic onset [6]. However, the present results provide an important first prospective insight into HPA axis activity and psychosocial stress before and at the time of tic onset.

## 5. Conclusions

In sum, the present study provides the first prospective data on HPA axis activity and perceived stress prior to onset. Results show a non-linear increasing course of HPA axis activity in the year before the onset of tics and, in particular, a higher hair cortisol concentration within the five months before tic onset, indicating that an increase in stress over months predicts a subsequent tic onset rather than an acute increase. Furthermore, the present results offer an indication that a dysregulation within the HPA axis might be a factor in the mechanism of tic onset, whereas it is still unclear whether a microbiologically triggered immune response, an infection-triggered stress response, or perhaps fronto-subcortical networks with immune-mediated insult might be causal for the dysregulation (for an overview, see [14,15]). To date, there is a lack of research on how dysregulation within the HPA axis may be related to tics and hippocampal volume. Further research is needed on the role of stress and the regulation of the HPA axis in individuals with CTDs or at high risk of developing tics. In particular, the interaction and mediation effects of HCC and self-reported perceived stress in relation to the onset of tics should be investigated in future research. For example, future studies could use wearables and apps to collect real-time data on physiological and psychosocial stress by leveraging more extensive and fine-tuned data. Such technologies may also be used in clinical practice as low-threshold instruments to monitor stress reports and markers in high-risk individuals. In addition, prevention programmes for high-risk individuals such as mindfulness-based stress reduction could be implemented in clinical practice; mindfulness-based stress reduction appears to improve the severity of tics and tic-related impairments in people with CTDs [48]. Thus, not only children with CTDs but also their siblings could benefit from a mindfulness-based stress reduction programme, which could be extended by a mindfulness-based parenting programme.

## Figures and Tables

**Table 1:** Characteristics and differences between Onset+ and Onset− children of the total study sample and the subsample used for binary logistic regression analyses.

	Onset+	Onset−	Test Statistic	*p*-Value	Effect Size
Children included in the present study					
*n*	54	177			
Sex, male, *n* (%)	33 (61%)	67 (38%)	X^2^ (1) = 9.12	*p* = 0.003	φ = 0.19
Highest education parents, *Md*	5	5	X^2^ (4) = 1.92	*p* = 0.75	V = 0.09
ADHD (DSM-IV-TR), *n* (%)	6 (11%)	13 (7%)	X^2^ (1) = 0.63	*p* = 0.43	φ = 0.05
Age, *M* (SD)	6.61 (1.83)	6.69 (2.16)	*t* (229) = −0.25	*p* = 0.80	*d* = 0.04
Study duration in weeks, *M* (SD)	51.41 (40.10)	84.68 (53.07)	*t* (114.88) = −4.92	*p* < 0.001	*d* = −0.66
Sample used for binary logistic regression					
*n*	13	59			
Sex, male, *n* (%)	8 (62%)	17 (29%)	X^2^ (1) = 5.03	*p* = 0.03	φ = 0.26
Highest education parents, *Md*	4	4	X^2^ (4) = 0.15	*p* = 0.99	V = 0.05
ADHD (DSM-IV-TR), *n* (%)	0 (0%)	7 (12%)	X^2^ (1) = 1.71	*p* = 0.19	φ = 0.15
Age, *M* (SD)	6.94 (1.89)	7.27 (2.32)	*t* (70) = −0.48	*p* = 0.63	*d* = 0.15
Weeks between T1 and T2, *M* (SD) Range	17.24 (2.05) 14–20	17.02 (1.87) 12–22	*t* (70) = 0.37	*p* = 0.72	*d* = 0.11
Weeks between T2 and T3, *M* (SD) Range	14.15 (4.47) 6–20	16.63 (2.06) 10–23	*t* (13.14) = −1.95	*p* = 0.07	*d* = −0.93

Note. ADHD = attention-deficit/hyperactivity disorder, includes all children with inattentive or hyperactive or combined type. Highest parental education level according to six categories: 1 = under seven years of schooling, 2 = Junior High School/7th–9th grade, 3 = General Certificate of Secondary Education or high school diploma, 4 = A levels or two-year college degree, 5 = four-year college/university degree, and 6 = post-graduate/graduate/professional degree. Onset+ = children with the onset of tics during study participation; Onset− = children without the onset of tics during study participation. The time ranges of T1, T2, and T3 are described in Table 2.

**Table 2:** Description of visits used for the binary logistic regression analyses of stress measures (HCC and PSS-10) between the children with tic onset (Onset+) and the children without tic (Onset−).

	Onset+ *n* = 13	Onset− *n* = 59	Test Statistic	*p*-Value	Effect Size
T1					
Description of the visit and time range	Second last visit before tic onset with valid hair sample and PSS-10 (visit occurred 6–9 months before onset)	Baseline visit with a valid hair sample			
HCC, *M* (SD) ^1^	4.93 (5.22)	2.94 (3.84)	*t* (70) = 1.43	*p* = 0.16	*d* = 0.44
PSS-10, *M* (SD) ^1^	11.38 (7.14)	10.38 (6.56)	*t* (70) = 0.45	*p* = 0.66	*d* = 0.14
T2					
Description of the visit and time range	Last visit before tic onset with valid hair sample and PSS-10 (visit occurred 2–5 months before onset)	First follow-up visit with a valid hair sample			
HCC, *M* (SD) ^1^	5.62 (5.48)	2.26 (1.94)	*t* (70) = 2.63	*p* = 0.01 ^2^	*d* = 0.81
PSS-10, *M* (SD) ^1^	11.23 (6.62)	10.70 (7.98)	*t* (70) = 0.32	*p* = 0.75	*d* = 0.10
T3					
Description of the visit and time range	At onset with valid hair sample and PSS-10	Second follow-up visit with a valid hair sample			
HCC, *M* (SD) ^1^	3.31 (2.71)	2.61 (3.24)	*t* (70) = 1.16	*p* = 0.25	*d* = 0.36
PSS-10, *M* (SD) ^1^	11.23 (6.62)	10.70 (7.98)	*t* (70) = 0.67	*p* = 0.51	*d* = 0.20

Note. HCC = hair cortisol concentration; PSS-10 = parent-on-child-reported Perceived Stress Scale. Note that only subjects with three consecutive visits of available HCC and perceived stress were included in these groups. ^1^ for the mean and standard deviation, untransformed values were displayed, while test statistics were calculated by transformed values. ^2^ result was significant after FDR correction (10%), six comparisons.

## Data Availability

The datasets used and/or analysed during the current study are available from the corresponding author upon reasonable request.

## Supplementary Materials

The following supporting information can be downloaded at: https://www.mdpi.com/article/10.3390/biomedicines11061561/s1, Table S1: Selection of the sample for analysis; Figure S1: EMTICS Onset−cohort study procedures flow chart (regular three-year study period); Table S2: Coefficients of the generalized additive models to test the moderating effect of Onset on the relationship between time (Weeks to Onset/Last Visit) and HCC/PSS-10; Table S3. Coefficients of the generalized additive models to test the moderating effect of Onset on the relationship between time (Weeks to Onset/Last Visit) and HCC/PSS-10 after excluding of one sibling in each sibling-pair; Table S4: Coefficients of the generalized additive models to test the moderating effect of Onset on the relationship between time (Weeks to Onset/Last Visit) and HCC/PSS-10 with a case-control matched sample (without sibling-pairs).

## Appendix A

**EMTICS GROUP AUTHORSHIP**: Alan Apter, Noa Benaroya-Milshtein, Francesco Cardona, Tammy Hedderly, Chaim Huyser, Davide, Martino, Pablo Mir, Astrid Morer Liñan, Norbert Muller, Kirsten R Müller-Vahl, Alexander Münchau, Kerstin J. Plessen, Cesare Porcelli, Renata Rizzo, Anette Schrag, Zsanett Tarnok, Susanne Walitza, Julie Hagstrøm, Isobel Heyman, Valentina Baglioni, Juliane Ball, Emese Bognar, Marta Correa Vela, Maria Cristina Ferro, Carolin Fremer, Blanca Garcia-Delgar, Mariangela Gulisano, Marcos Madruga-Garrido, Peter Nagy, Valeria Neri, Daphna Ruhrman, Liselotte Skov, Tamar Steinberg, Friederike Tagwerker Gloor, and Victoria L. Turner.

## References

[1] Cubo E., Trejo Gabriel y Galán J.M., Villaverde V.A., Sáez Velasco S., Delgado Benito V., Vicente Macarrón J., Guevara J.C., Louis E.D., Benito-León J. (2011). Prevalence of Tics in Schoolchildren in Central Spain: A Population-Based Study. Pediatr. Neurol..

[2] Kurlan R., Como P.G., Miller B., Palumbo D., Deeley C., Andresen E.M., Eapen S., McDermott M.P. (2002). The Behavioral Spectrum of Tic Disorders: A Community-Based Study. Neurology.

[3] Snider L.A., Seligman L.D., Ketchen B.R., Levitt S.J., Bates L.R., Garvey M.A., Swedo S.E. (2002). Tics and Problem Behaviors in Schoolchildren: Prevalence, Characterization, and Associations. Pediatrics.

[4] Robertson M.M., Eapen V., Singer H.S., Martino D., Scharf J.M., Paschou P., Roessner V., Woods D.W., Hariz M., Mathews C.A. (2017). Gilles de La Tourette Syndrome. Nat. Rev. Dis. Prim..

[5] Knight T., Steeves T., Day L., Lowerison M., Jette N., Pringsheim T. (2012). Prevalence of Tic Disorders: A Systematic Review and Meta-Analysis. Pediatr. Neurol..

[6] Openneer T.J.C., Huyser C., Martino D., Schrag A., Group E.C., Hoekstra P.J., Dietrich A. (2022). Clinical Precursors of Tics: An EMTICS Study. J. Child Psychol. Psychiatry.

[7] Bornstein R.A., Stefl M.E., Hammond L. (1990). A Survey of Tourette Syndrome Patients and Their Families: The 1987 Ohio Tourette Survey. J. Neuropsychiatry Clin. Neurosci..

[8] Horesh N., Zimmerman S., Steinberg T., Yagan H., Apter A. (2008). Is Onset of Tourette Syndrome Influenced by Life Events?. J. Neural Transm..

[9] Buonsenso D., Rose C.D., Mariotti P. (2021). Children Experienced New or Worsening Tic Issues When They Were Separated from Their Parents during the Italian COVID-19 Lockdown. Acta Paediatr..

[10] Jones H.F., Han V.X., Patel S., Gloss B.S., Soler N., Ho A., Sharma S., Kothur K., Nosadini M., Wienholt L. (2021). Maternal Autoimmunity and Inflammation Are Associated with Childhood Tics and Obsessive-Compulsive Disorder: Transcriptomic Data Show Common Enriched Innate Immune Pathways. Brain Behav. Immun..

[11] Lazarus R.S., Folkman S. (1984). Stress, Appraisal, and Coping.

[12] Steudte-Schmiedgen S., Stalder T., Schönfeld S., Wittchen H.-U., Trautmann S., Alexander N., Miller R., Kirschbaum C. (2015). Hair Cortisol Concentrations and Cortisol Stress Reactivity Predict PTSD Symptom Increase after Trauma Exposure during Military Deployment. Psychoneuroendocrinology.

[13] Caparros-Gonzalez R.A., Romero-Gonzalez B., Strivens-Vilchez H., Gonzalez-Perez R., Martinez-Augustin O., Peralta-Ramirez M.I. (2017). Hair Cortisol Levels, Psychological Stress and Psychopathological Symptoms as Predictors of Postpartum Depression. PLoS ONE.

[14] Ahrens K.F., Neumann R.J., von Werthern N.M., Kranz T.M., Kollmann B., Mattes B., Puhlmann L.M.C., Weichert D., Lutz B., Basten U. (2022). Association of Polygenic Risk Scores and Hair Cortisol with Mental Health Trajectories during COVID Lockdown. Transl. Psychiatry.

[15] Frost A., Hagaman A., Baranov V., Chung E.O., Bhalotra S., Sikander S., Maselko J. (2022). Non-Linear Associations between HPA Axis Activity during Infancy and Mental Health Difficulties during Early Childhood among Children in Rural Pakistan. Dev. Psychopathol..

[16] Ford J.L., Boch S.J., Browning C.R. (2019). Hair Cortisol and Depressive Symptoms in Youth: An Investigation of Curvilinear Relationships. Psychoneuroendocrinology.

[17] Corbett B.A., Mendoza S.P., Baym C.L., Bunge S.A., Levine S. (2008). Examining Cortisol Rhythmicity and Responsivity to Stress in Children with Tourette Syndrome. Psychoneuroendocrinology.

[18] Chappell P., Riddle M., Anderson G., Scahill L., Hardin M., Walker D., Cohen D., Leckman J. (1994). Enhanced Stress Responsivity of Tourette Syndrome Patients Undergoing Lumbar Puncture. Biol. Psychiatry.

[19] Chappell P., Leckman J., Goodman W., Bissette G., Pauls D., Anderson G., Riddle M., Scahill L., McDougle C., Cohen D. (1996). Elevated Cerebrospinal Fluid Corticotropin-Releasing Ractor in Tourette’s Syndrome: Comparison to Obsessive Compulsive Disorder and Normal Controls. Biol. Psychiatry.

[20] Buse J., Rothe J., Uhlmann A., Bodmer B., Kirschbaum C., Hoekstra P.J., Dietrich A., Roessner V., Apter A., Baglioni V. (2021). Hair Cortisol-a Stress Marker in Children and Adolescents with Chronic Tic Disorders? A Large European Cross-Sectional Study. Eur. Child Adolesc. Psychiatry.

[21] Browne H.A., Hansen S.N., Buxbaum J.D., Gair S.L., Nissen J.B., Nikolajsen K.H., Schendel D.E., Reichenberg A., Parner E.T., Grice D.E. (2015). Familial Clustering of Tic Disorders and Obsessive-Compulsive Disorder. JAMA Psychiatry.

[22] Schrag A., Martino D., Apter A., Ball J., Bartolini E., Benaroya-Milshtein N., Buttiglione M., Cardona F., Creti R., Efstratiou A. (2019). European Multicentre Tics in Children Studies (EMTICS): Protocol for Two Cohort Studies to Assess Risk Factors for Tic Onset and Exacerbation in Children and Adolescents. Eur. Child Adolesc. Psychiatry.

[23] Stalder T., Kirschbaum C. (2012). Analysis of Cortisol in Hair—State of the Art and Future Directions. Brain Behav. Immun..

[24] Dettenborn L., Tietze A., Kirschbaum C., Stalder T. (2012). The Assessment of Cortisol in Human Hair: Associations with Sociodemographic Variables and Potential Confounders. Stress.

[25] Cohen S., Kamarck T., Mermelstein R. (1983). A Global Measure of Perceived Stress. J. Health Soc. Behav..

[26] Lee E.-H. (2012). Review of the Psychometric Evidence of the Perceived Stress Scale. Asian Nurs. Res..

[27] Findley D.B., Leckman J.F., Katsovich L., Lin H., Zhang H., Grantz H., Otka J., Lombroso P.J., King R.A. (2003). Development of the Yale Children’s Global Stress Index (YCGSI) and Its Application in Children and Adolescents With Tourette’s Syndrome and Obsessive-Compulsive Disorder. J. Am. Acad. Child Adolesc. Psychiatry.

[28] Benjamini Y., Yekutieli D. (2001). The Control of the False Discovery Rate in Multiple Testing under Dependency. Ann. Stat..

[29] Cohen J. (2013). Statistical Power Analysis for the Behavioral Sciences.

[30] Hastie T., Tibshirani R., Friedman J.H., Friedman J.H. (2009). The Elements of Statistical Learning: Data Mining, Inference, and Prediction.

[31] Wood S.N. (2011). Fast Stable Restricted Maximum Likelihood and Marginal Likelihood Estimation of Semiparametric Generalized Linear Models. J. R. Stat. Soc. Ser. B (Stat. Methodol.).

[32] Osborne J. (2019). Improving Your Data Transformations: Applying the Box-Cox Transformation. Pract. Assess. Res. Eval..

[33] Stalder T., Steudte-Schmiedgen S., Alexander N., Klucken T., Vater A., Wichmann S., Kirschbaum C., Miller R. (2017). Stress-Related and Basic Determinants of Hair Cortisol in Humans: A Meta-Analysis. Psychoneuroendocrinology.

[34] Holm J.E., Holroyd K.A. (1992). The Daily Hassles Scale (Revised): Does It Measure Stress or Symptoms?. Behav. Assess..

[35] Weckesser L.J., Dietz F., Schmidt K., Grass J., Kirschbaum C., Miller R. (2019). The Psychometric Properties and Temporal Dynamics of Subjective Stress, Retrospectively Assessed by Different Informants and Questionnaires, and Hair Cortisol Concentrations. Sci. Rep..

[36] Martino D., Dale R.C., Gilbert D.L., Giovannoni G., Leckman J.F. (2009). Immunopathogenic Mechanisms in Tourette Syndrome: A Critical Review. Mov. Disord..

[37] Tao Y., Xu P., Zhu W., Chen Z., Tao X., Liu J., Xue Z., Zhu T., Jiang P. (2022). Changes of Cytokines in Children With Tic Disorder. Front. Neurol..

[38] Yeon S., Lee J.H., Kang D., Bae H., Lee K.Y., Jin S., Kim J.R., Jung Y.W., Park T.W. (2017). A Cytokine Study of Pediatric Tourette’s Disorder without Obsessive Compulsive Disorder. Psychiatry Res..

[39] Leckman J.F., Katsovich L., Kawikova I., Lin H., Zhang H., Krönig H., Morshed S., Parveen S., Grantz H., Lombroso P.J. (2005). Increased Serum Levels of Interleukin-12 and Tumor Necrosis Factor-Alpha in Tourette’s Syndrome. Biol. Psychiatry.

[40] Lemos J.C., Wanat M.J., Smith J.S., Reyes B.A.S., Hollon N.G., Van Bockstaele E.J., Chavkin C., Phillips P.E.M. (2012). Severe Stress Switches CRF Action in the Nucleus Accumbens from Appetitive to Aversive. Nature.

[41] Payer D., Williams B., Mansouri E., Stevanovski S., Nakajima S., Le Foll B., Kish S., Houle S., Mizrahi R., George S.R. (2017). Corticotropin-Releasing Hormone and Dopamine Release in Healthy Individuals. Psychoneuroendocrinology.

[42] Ganos C., Roessner V., Münchau A. (2013). The Functional Anatomy of Gilles de La Tourette Syndrome. Neurosci. Biobehav. Rev..

[43] Buse J., Kirschbaum C., Leckman J.F., Münchau A., Roessner V. (2014). The Modulating Role of Stress in the Onset and Course of Tourette’s Syndrome: A Review. Behav. Modif..

[44] Hsu C.-J., Wong L.-C., Lee W.-T. (2021). Immunological Dysfunction in Tourette Syndrome and Related Disorders. Int. J. Mol. Sci..

[45] Cox J.H., Seri S., Cavanna A.E. (2018). Sensory Aspects of Tourette Syndrome. Neurosci. Biobehav. Rev..

[46] Belluscio B.A., Jin L., Watters V., Lee T.H., Hallett M. (2011). Sensory Sensitivity to External Stimuli in Tourette Syndrome Patients. Mov. Disord..

[47] Buse J., Beste C., Herrmann E., Roessner V. (2016). Neural Correlates of Altered Sensorimotor Gating in Boys with Tourette Syndrome: A Combined EMG/FMRI Study. World J. Biol. Psychiatry.

[48] Reese H.E., Vallejo Z., Rasmussen J., Crowe K., Rosenfield E., Wilhelm S. (2015). Mindfulness-Based Stress Reduction for Tourette Syndrome and Chronic Tic Disorder: A Pilot Study. J. Psychosom. Res..

