# Supplementary material for: Hair Cortisol and Perceived Stress—Predictors for the Onset of Tics? A European Longitudinal Study on High-Risk Children
Source: Biomedicines. 2023 May 27;11(6):1561. doi: 10.3390/biomedicines11061561 (PMC10295562; doi:10.3390/biomedicines11061561)
Supplement: Supplementary file 1 [file biomedicines-11-01561-s001.zip › biomedicines-2271929-supplementary.pdf]

biomedicines

MDPI

Supplementary Material

Table S1. Selection of the sample for analysis.

Selection	Sample size
Total sample of the ONSET arm	259
Participants with onset of tics	61
Participants without onset of tics	198
Participants with onset of tics & OCD symptomatology during baseline	7
Participants without onset of tics & OCD symptomatology during baseline	17
Participants with onset of tics & trichotillomania during baseline	0
Participants without onset of tics & trichotillomania during baseline	4
participants of the ONSET arm met all inclusion criteria	231
Participants with onset of tics met all inclusion criteria	54
Participants with onset of tics & at least one valid HS	42
Participants with onset of tics & valid HS at Onset-Visit (T3)	31
Participants with onset of tics, valid HS at Onset-Visit (T3) & valid HS 2-5 month before Onset-Visit (T2)	25
Participants with onset of tics, valid HS at Onset-Visit (T3), valid HS 2-5 month before Onset-Visit (T2) & valid HS 6-9 month before Onset-Visit (T1)	16
Participants with onset of tics, valid HS at Onset-Visit (T3), valid HS 2-5 month before Onset-Visit (T2) & valid HS 6-9 month before Onset-Visit (T1) & complete PSS-10 data for all three visits	13
Participants without onset of tics met all inclusion criteria	177
Participants without onset of tics & at least one valid HS	152
Participants without onset of tics & with valid HS at Baseline (T1)	124
Participants without onset of tics, with valid HS at Baseline (T1) & valid HS at first follow-up (T2, at most 24 weeks after baseline visit)	89
Participants without onset of tics, with valid HS at Baseline (T1), valid HS at first follow-up (T2, at most 24 weeks after baseline visit) & valid HS at second follow-up (T3, at most 24 weeks after first follow-up)	68
Participants without onset of tics, with valid HS at Baseline (T1), valid HS at first follow-up (T2, at most 24 weeks after baseline visit) & valid HS at second follow-up (T3, at most 24 weeks after first follow-up) & complete PSS-10 data for all three visits	59

Note. HS = hair sample.

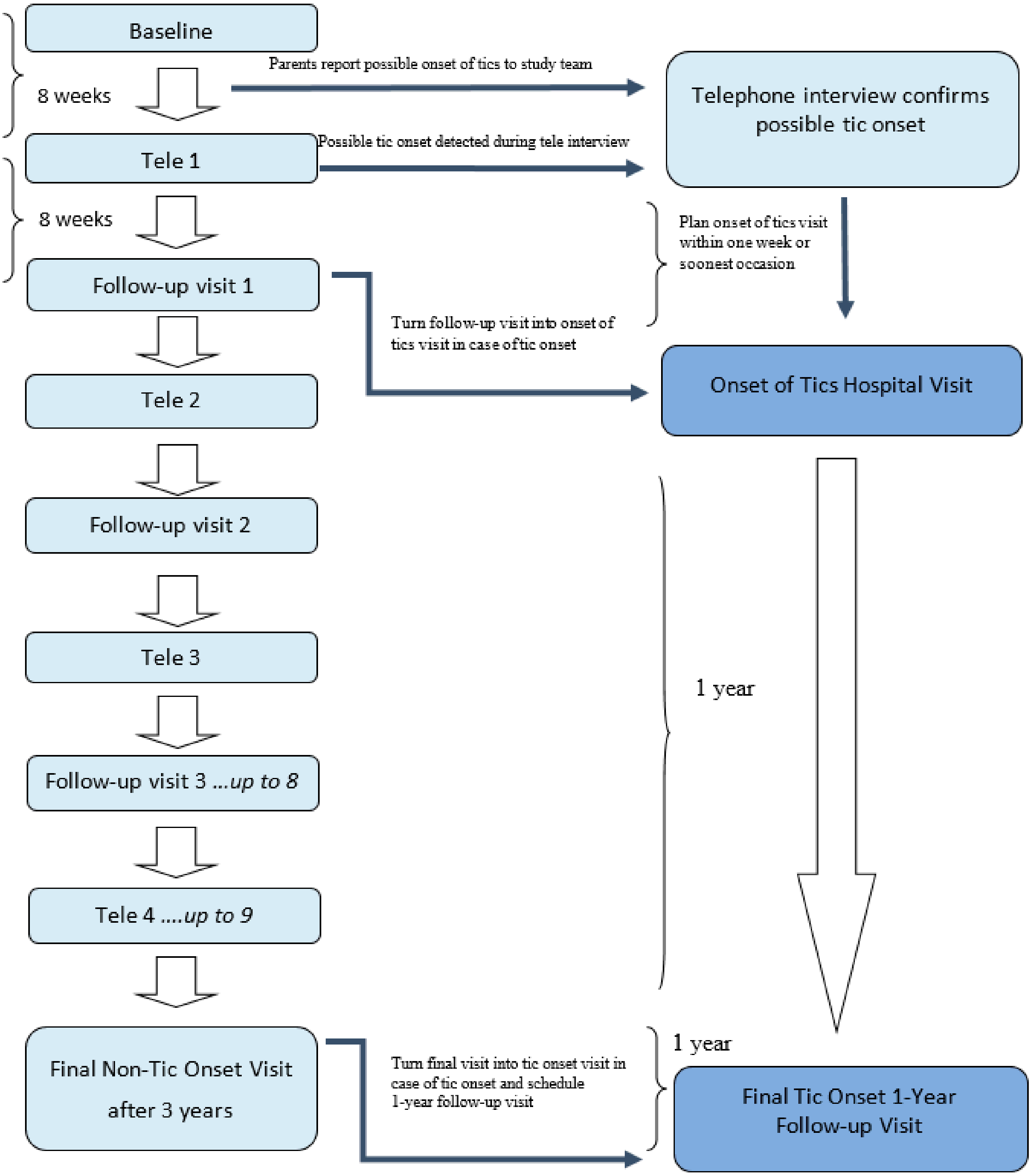

Figure S1. EMTICS ONSET cohort study procedures flow chart (regular 3-year study period). *Note:* Tele=telephone interview. If no tic onset was detected and confirmed then the original assessment schedule (left side) was retained. After an onset of tics hospital visit all further assessments were discarded, except for a 1-year follow-up visit. The minimum study period was one year, tic onset at the final visit increased the maximum study period to 4 years.

Table S2. Coefficients of the generalized additive models to test the moderating effect of Onset on the relationship between time (Weeks to Onset/Last Visit) and HCC/PSS-10.

**HCC**	**Estimate**	**SE**	***t***	***p***
(Intercept)	.84	.05	15.60	< .001
Age	-.01	.01	-1.16	.25
Female sex (reference: male sex)	.03	.03	1.24	.22
Onset- (reference: Onset+)	.01	.04	.17	.87
**Smooth terms**	**edf**	**Ref.df**	**F**	**p**
s(Time): Onset+	2.25	2.81	2.12	.07
s(Time): Onset-	1.00	1.00	3.06	.08

**PSS-10**	**Estimate**	**SE**	***t***	***p***
(Intercept)	7.14	.39	18.18	< .001
Age	-.09	.04	-2.08	.04
Female sex (reference: male sex)	-.17	.20	-.82	.41
Onset- (reference: Onset+)	-.36	.28	-1.28	.20
**Smooth terms**	**edf**	**Ref.df**	***F***	***p***
s(Time): Onset+	1.00	1.01	2.95	.09
s(Time): Onset-	1.00	1.00	3.70	.05

Note. The HCC model includes 128 hair samples of Onset+ participants and 637 of Onset- participants. The PSS-10 model includes 190 PSS-10 values of Onset+ participants and 829 of Onset- participants.

Table S3. Coefficients of the generalized additive models to test the moderating effect of Onset on the relationship between time (Weeks to Onset/Last Visit) and HCC/PSS-10 after excluding of one sibling in each sibling-pair.

**HCC**	**Estimate**	**SE**	***t***	***p***
(Intercept)	.83	.06	14.38	< .001
Age	-.01	.01	.-1.18	.24
Female sex (reference: male sex)	.07	.03	2.43	.02
Onset- (reference: Onset+)	.01	.04	.38	.70
**Smooth terms**	**edf**	**Ref.df**	**F**	**p**
s(Time): Onset+	2.23	2.78	2.08	.08
s(Time): Onset-	1.00	1.00	.06	.81

**PSS-10**	**Estimate**	**SE**	***t***	***p***
(Intercept)	6.98	.42	16.44	< .001
Age	-.07	.05	-1.41	.16
Female sex (reference: male sex)	-.08	.22	-.36	.72
Onset- (reference: Onset+)	-.41	.29	-1.43	.15
**Smooth terms**	**edf**	**Ref.df**	***F***	***p***
s(Time): Onset+	1.00	1.00	2.91	.09
s(Time): Onset-	1.00	1.00	2.30	.13

Note. The HCC model includes 128 hair samples of Onset+ participants and 541 of Onset- participants. The PSS-10 model includes 182 PSS-10 values of Onset+ participants and 714 of Onset- participants.

Table S4. Coefficients of the generalized additive models to test the moderating effect of Onset on the relationship between time (Weeks to Onset/Last Visit) and HCC/PSS-10 with a case-control matched sample (without sibling-pairs).

**HCC**	**Estimate**	**SE**	***t***	***p***
(Intercept)	.92	.10	9.49	< .001
Age	-.02	.01	-1.64	.10
Female sex (reference: male sex)	.11	.05	2.14	.03
Onset- (reference: Onset+)	-.01	.05	-.30	.76
**Smooth terms**	**edf**	**Ref.df**	**F**	**p**
s(Time): Onset+	2.18	2.71	2.07	.08
s(Time): Onset-	1.00	1.00	.00	.99

**PSS-10**	**Estimate**	**SE**	***t***	***p***
(Intercept)	6.87	.71	9.64	< .001
Age	-.05	.10	-.54	.59
Female sex (reference: male sex)	.04	.37	.11	.91
Onset- (reference: Onset+)	-.50	.36	-1.40	.16
**Smooth terms**	**edf**	**Ref.df**	***F***	***p***
s(Time): Onset+	1.00	1.00	2.49	.12
s(Time): Onset-	1.00	1.00	1.21	.27

Note. The HCC model includes 128 hair samples of Onset+ participants and 128 of Onset- participants. The PSS-10 model includes 182 PSS-10 values of Onset+ participants and 182 of Onset- participants.
